# The Cost-effectiveness of a Mass Media Campaign to Promote Smartphone Apps for Weight Loss: Updated Modeling Study

**DOI:** 10.2196/29291

**Published:** 2022-04-19

**Authors:** Amanda C Jones, Leah Grout, Nick Wilson, Nhung Nghiem, Christine Cleghorn

**Affiliations:** 1 Department of Public Health University of Otago Wellington New Zealand

**Keywords:** mass media, smartphone apps, weight loss, cost-effectiveness, simulation modeling, health equity, mobile phone

## Abstract

**Background:**

Evidence suggests that smartphone apps can be effective in the self-management of weight. Given the low cost, broad reach, and apparent effectiveness of weight loss apps, governments may seek to encourage their uptake as a tool to reduce excess weight in the population. Mass media campaigns are 1 mechanism for promoting app use. However, the cost and potential cost-effectiveness are important considerations.

**Objective:**

The aim of our study was to use modeling to assess the health impacts, health system costs, cost-effectiveness, and health equity of a mass media campaign to promote high-quality smartphone apps for weight loss in New Zealand.

**Methods:**

We used an established proportional multistate life table model that simulates the 2011 New Zealand adult population over the lifetime, subgrouped by age, sex, and ethnicity (Māori [Indigenous] or non-Māori). The risk factor was BMI. The model compared business as usual to a one-off mass media campaign intervention, which included the pooled effect size from a recent meta-analysis of smartphone weight loss apps. The resulting impact on BMI and BMI-related diseases was captured through changes in health gain (quality-adjusted life years) and in health system costs. The difference in total health system costs was the net sum of intervention costs and downstream cost offsets because of altered disease rates. An annual discount rate of 3% was applied to health gains and health system costs. Multiple scenarios and sensitivity analyses were conducted, including an equity adjustment.

**Results:**

Across the remaining lifetime of the modeled 2011 New Zealand population, the mass media campaign to promote weight loss app use had an estimated overall health gain of 181 (95% uncertainty interval 113-270) quality-adjusted life years and health care costs of –NZ $606,000 (–US $408,000; 95% uncertainty interval –NZ $2,540,000 [–US $1,709,000] to NZ $907,000 [US $610,000]). The mean health care costs were negative, representing overall savings to the health system. Across the outcomes examined in this study, the modeled mass media campaign to promote weight loss apps among the general population would be expected to provide higher per capita health gain for Māori and hence reduce health inequities arising from high BMI, assuming that the intervention would be as effective for Māori as it is for non-Māori.

**Conclusions:**

A modeled mass media campaign to encourage the adoption of smartphone apps to promote weight loss among the New Zealand adult population is expected to yield an overall gain in health and to be cost-saving to the health system. Although other interventions in the nutrition and physical activity space are even more beneficial to health and produce larger cost savings (eg, fiscal policies and food reformulation), governments may choose to include strategies to promote health app use as complementary measures.

## Introduction

### Background

The obesogenic food environment and unhealthy dietary patterns have led to overweight and obesity becoming a critical public health problem [1,2]. These risk factors result in numerous health conditions, including diabetes, cardiovascular disease, and certain forms of cancer [1,2]. Overweight and obesity are the fifth highest risk factor for global mortality, corresponding to at least 2.8 million deaths each year [1]. Elevated BMI levels also pose a substantial economic burden, with obesity alone directly accounting for an estimated 0.7% to 2.8% of national health care expenditures among a wide range of countries [3].

Although modifying the food and physical activity environment is critical, addressing unhealthy dietary patterns and insufficient physical activity (and therefore overweight and obesity [4]) in individuals is also important. Even modest weight loss can yield substantial health benefits [5,6], especially when distributed across the population level. There has been an increase in the use of mobile health (mHealth) tools for addressing weight loss goals [7,8]. The widespread use of mobile phones makes mHealth interventions easily scalable to a broader population [9,10], and as a result, mHealth interventions are increasingly considered tools for weight loss in individuals. Although there are numerous types of mHealth tools (eg, PDAs, iPods, and MP3 players) and services (eg, health call centers, appointment reminders, health surveys and data collection, and mobile patient records [11]), smartphone apps have been identified as particularly popular among the general population and potentially effective at supporting weight management [7,9,12].

The use of health apps is increasing, with a reported 50% of smartphone users having ever downloaded a health app [13,14]. The most popular health apps are typically for diet and physical activity tracking, weight management, and adherence to medication [13,15]. Weight loss apps may be especially useful for circumstances where face-to-face weight loss treatments are not possible or not preferred [16]. Smartphone apps are generally considered easy to use and to be helpful in pursuing weight loss goals by many patients [7], even for older participants [12].

Reviews have found that mHealth interventions can be more effective than non-mHealth interventions at inducing weight loss and improving diet and physical activity [17]; mHealth interventions also promote adherence to weight loss behaviors [9]. Even when mHealth apps produce outcomes equivalent to those of traditional interventions, their broad accessibility makes them a valuable tool [7]. However, there are limitations to the effectiveness of app use. For instance, engagement with apps declines over time [7,18], especially for dietary tracking [19,20]. Researchers estimate that a quarter of mHealth apps are only used once after download, and most individuals stop using mHealth tools before the fifth interaction [21,22]. However, other evidence suggests that apps are most effective for weight loss management when certain characteristics are incorporated [7], such as self-monitoring of physical activity and diet, reminders for app use, and social interaction with peers [7,23,24]. Generally, higher adherence to self-monitoring is associated with improved weight loss outcomes [10,25].

Given the low cost, broad reach, and apparent effectiveness of apps at promoting weight loss, governments may seek to encourage the uptake of such apps as an opportunity for reducing excess weight among the population. For example, in the United Kingdom, the National Health Service has developed a free 12-week diet and exercise plan that is available as an app [26] that incorporates a mass media campaign to promote use [27]. Such campaigns may be an important intervention component to increase the use of effective weight loss apps among the population and stimulate reductions in BMI. Evidence indicates that health mass media interventions can effectively encourage the use of health support resources [28,29] and influence public dietary behaviors [30-35].

The cost and potential cost-effectiveness are important considerations when governments are selecting among obesity reduction interventions, including the use of mass media campaigns. Research by Cleghorn et al [36] used health economic simulation modeling to assess the cost-effectiveness of a hypothetical mass media campaign in New Zealand that promotes the uptake of weight loss mHealth technologies. Along with numerous other parameters, the authors used the results of a 2014 meta-analysis of mobile device interventions to quantify how much weight loss could occur [17]. The authors found that such a campaign was not cost-effective in the base case analysis [36]. Another study using similar methods assessed the potential of a mass media campaign to promote smartphone apps for physical activity and found that it was unlikely to be cost-effective at the population level, although the health impact and cost-effectiveness estimates were highly sensitive to assumptions around long-term adherence [37]. The evidence on the cost-effectiveness of mass media campaigns is primarily from tobacco control [29], and previous research indicates that a mass media campaign for promoting smoking cessation apps is likely to be cost saving [38].

### Updated Estimates

Since the modeling conducted by Cleghorn et al [36] on the weight loss mHealth mass media campaign, there has been rapid growth in the literature on the effectiveness of weight loss apps, as well as changes in app technology and a higher uptake of smartphones. In this study, we use updated parameters that reflect these developments and recent data on app use over time, thereby providing updated estimates that reflect the current context. The setting for this study is New Zealand, a typical high-income country with high rates of overweight and obesity [39]. Our study uses a multistate life table modeling approach to assess the health impacts, health system costs, cost-effectiveness, and health equity of a mass media campaign to promote high-quality smartphone apps for weight loss in New Zealand.

## Methods

### Overview

We used an established proportional multistate life table model [40] that was parameterized with health data for the 2011 New Zealand adult population and simulates this population until death. The risk factor of interest was BMI; the simulation model designates the proportion of New Zealand adults with overweight or obesity (overweight: BMI 25 kg/m^2^ to <30 kg/m^2^; obesity: BMI ≥30 kg/m^2^). We compared a business-as-usual baseline analysis to an intervention base case analysis. The resulting impact on BMI and BMI-related diseases was captured through changes in health gain (as measured by quality-adjusted life years [QALYs]) and in health system costs (net sum of intervention costs and downstream cost offsets because of altered disease rates). The study used a health system perspective that focused on costs and benefits within the health system [41]. An annual discount rate of 3% was applied to health gains and health system costs, consistent with established New Zealand modeling protocols [41] and health economic expert recommendations [42]. Discounting is a standard practice in economic evaluation to account for preferences toward present benefits over future benefits [41]. Full details on the model are documented elsewhere [40].

### Intervention Pathway

**Figure 1:**
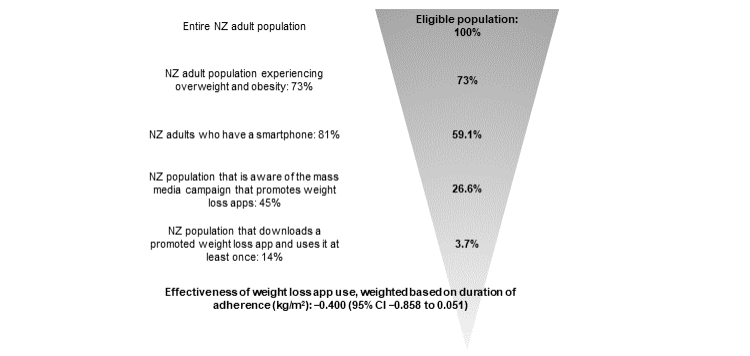
Flowchart of intervention conceptualization. NZ: New Zealand.

The modeled intervention was a one-off mass media campaign among the New Zealand population stimulating the uptake and use of a smartphone app for weight loss that effectively promotes weight loss. The pathway from the intervention’s implementation to impact is detailed in Figure 1. The proportion of the New Zealand population that would experience weight loss was calculated as follows: out of the New Zealand adult population, the intervention was applicable to only New Zealand adults aged >18 years with overweight or obesity (73% of the total adult population). Using recent metrics on New Zealand smartphone ownership, 81% of New Zealand adults own a smartphone [43] (eligible population reduced to 59.1%). To model the reach of the mass media campaign, we used the results reported by Kite et al [44] of an obesity-prevention mass media campaign called Make Healthy Normal that was run over 1 year by the New South Wales (NSW) government in Australia. Recognition of the campaign was 45% among sampled adults aged ≥18 years after completion of the campaign, which consisted of television commercials, community events, press, out-of-home (eg, billboards) and web-based advertising, public relations, a website, and social media [44]. We identified other studies that evaluated recognition of mass media campaigns [30,32]. However, the NSW study by Kite et al [44] was preferred because it evaluated a health campaign that we could verify was resourced similarly to the high level of costs associated with our modeled mass media campaign: Make Healthy Normal campaign NZ $0.37 (US $0.25) per capita of the NSW population [44] and modeled campaign NZ $0.66 (US $0.44) per capita of the New Zealand population. As the New Zealand modeled campaign involved a higher cost per capita than the NSW campaign, in sensitivity analysis we modeled a scenario where this greater resourcing produced an enhanced mass media campaign that achieved wider recognition than the NSW campaign. On the basis of the rate of campaign recognition, the eligible population reduced to 26.6% of the total adult population. We found limited published research on the degree to which government-led mass media campaigns can stimulate health app use. After a search of academic and gray literature, we identified only 1 study that quantified a relationship between a mass media campaign to promote health app use and subsequent health app use [45]. In the study, the authors found that 14% of the surveyed respondents reported *taking an action* after a UK-based mass media campaign to promote an uptake of a health app [45]. Assuming that this *action* was to download and use the app at least once, we modeled app downloads and use as 14% of the New Zealand adult population. The final eligible population that would use the app and experience weight loss was 3.7% of the New Zealand adult population.

### Weight Loss App Effectiveness

We quantified the effectiveness of weight loss apps using the results of a recent meta-analysis by Islam et al [46]. The review’s authors examined randomized controlled trials and case-control studies published from 2000 to mid-2019 on mobile phone app interventions for reducing body weight (including BMI) and increasing physical activity among children and adults. We preferentially selected this meta-analysis for modeling over other reviews [9,47-53] because of the focus on mobile apps (over other mHealth or mobile phone–based interventions), selection of studies with control groups that received minimal or no interventions, preferred outcome (ie, BMI), meta-analyzed results, and recent publication date with latest available studies. In the 10 included studies that examined BMI, the proportion of male participants ranged from 0% to 15%. The mean age of the participants ranged from 12.7 years to 44.9 years. For the outcome of weight, Islam et al [46] reported finding that longer trials (>3 months) were associated with significantly greater weight loss than shorter trials (≤3 months), suggesting that greater app adherence (ie, longer use) is associated with greater weight loss than shorter app adherence. For the outcome of BMI, Islam et al [46] conducted an analysis of all included trials (pooled effect size –0.454, 95% CI –0.787 to –0.121) and did not conduct meta-analyses by trial duration subgroup (ie, ≤3 months vs >3 months). We calculated these pooled effect sizes using the study’s reported data (≤3 months –0.219, 95% CI –0.672 to 0.223; >3 months –0.609, 95% CI –1.072 to –0.146) [46]. In the >3-month subgroup, the maximum trial length was 9 months. We consulted evidence on weight loss app adherence from the UK study by Carter et al [54], which measured the proportion of users who adhered to app use for ≤3 months (53% of the users) and >3 months (47% of the users). We multiplied each pooled effect size by the proportion of users and summed to produce a weighted effect size of app effectiveness that accounted for variations in duration of app use:

E_weighted_= (E_≤3 months_ × >P_≤3 months_) + (E_>3 months_ × P_>3 months_)

where *E* is the effect size and *P* is the proportion of users. The resulting weighted estimate of weight loss app effectiveness was –0.400 kg/m^2^ (95% CI –0.858 to 0.051 kg/m^2^). Additional details on these decisions and steps are reported in Multimedia Appendix 1 [19,46,54].

### Intervention Modeling Approach

In our modeling, the business-as-usual baseline encapsulated the existing levels of dietary health promotion in New Zealand, including current promotion of weight loss apps, and the continuation of the current low or no app promotion environment. The costs of implementing the intervention are reported in Table 1, with further details regarding the methods used to scope these costs reported by Cleghorn et al [36].

Table 1 provides further detail on the inputs used to conceptualize the intervention pathway, along with the type of distribution modeled for each of these inputs. For the effectiveness of the weight loss app, CIs quantified the range of plausible values. As no measures of variance were available for the remaining inputs, we applied our established protocol and estimated an SD of either 5%, 10%, or 20% of the central value [56]. A value of 20% was used when there was deemed to be a high degree of uncertainty that could influence effectiveness and cost-effectiveness outcomes from the modeled intervention.

**Table 1:** Intervention parameters and uncertainty distributions.

Parameter	Value	Distribution	Source
Adult New Zealand population who own a smartphone, % (SD)	81 (5)	Beta	As reported by DataReportal based on Google Consumer Barometer data [43]
Adult New Zealand population who are assumed to be aware of a relevant mass media campaign, % (SD)	45 (20)	Beta	On the basis of an evaluation of an Australian obesity-prevention mass media campaign that measured the proportion of survey respondents who recognized the campaign [44]
Adult New Zealand population who were assumed to download and use a promoted weight loss app, % (SD)	14 (20)	Beta	On the basis of the proportion of survey respondents who reported *taking an action* after a UK-based mass media campaign to promote use of a health app [45]
Intervention BMI reduction for those who used the app (kg/m^2^; 95% CI)	–0.400 (–0.858 to 0.051)	Normal	The weighted results of studies included in the Islam et al [46] meta-analysis of smartphone app weight loss trials whereby the pooled effect sizes for interventions ≤3 months and interventions >3 months were weighted based on adherence rates at 3 months, obtained from Carter et al [54]
Assumed weight regain after delivery of the intervention (kg/m^2^ per month; SD %)	0.03 (20)	Log-normal	Meta-analysis of weight loss decay evidence from Dansinger et al [55], as used in the previous published work by Cleghorn et al [36]
Estimated cost of one-off 1-year national-level mass media campaign, NZ $ (US $; SD %)	2,883,000 (1,940,000; 20)	Gamma	As used in the previous published work by Cleghorn et al [36]. New Zealand data on the costs consist of identifying high-quality apps and a national mass media campaign across multiple media

### Multistate Life Table Model Overview

The multistate life table model consists of a main life table organized by age, sex (male or female), and ethnicity (Māori or non-Māori) and populated with all-cause mortality and morbidity rates for the 2011 New Zealand adult population. Parallel to this are life tables for each BMI-related disease where proportions of the simulated population are also modeled. Although the model includes a wide array of diet-related diseases [40], our intervention focused on weight loss and used only the disease tables for 14 BMI-related diseases: coronary heart disease, stroke, type 2 diabetes, osteoarthritis, and cancers (endometrial, kidney, liver, esophageal, pancreatic, thyroid, colorectal, breast, ovarian, and gallbladder).

### Mortality and Morbidity Modeling

Within the model, the proportions of the population in each disease table are a function of past and current rates of disease incidence, case fatality, and, for cancers only, remission, which are calculated at each annual time step. The model is populated with mean BMI values according to age, sex, and self-identified ethnicity measured in person during New Zealand’s most recent available national nutrition survey (New Zealand Adult Nutrition Survey 2008-2009) [57]. The mass media campaign intervention induces changes in BMI for a proportion of the New Zealand population. The effect of these BMI changes is combined with relative risks that capture the association between BMI and BMI-related disease outcomes to produce modified population impact fractions [40]. As the risk of BMI-related diseases decreases after implementation of the intervention, the population impact fractions modify disease incidence rates, resulting in changes to all-cause mortality and morbidity rates. Time lags to simulate the delay between when BMI change occurs and when changes in disease incidence across the population-risk distribution occur were built in for all conditions. Specifically, the change on disease rates was distributed over 0 to 5 years for cardiovascular diseases, diabetes, and osteoarthritis and over 10 to 30 years for cancers, with probabilistic uncertainty added in around these lag periods. Although there is evidence that mHealth interventions can result in changes in dietary intake beyond reduced weight loss, such as increased fruit and vegetable intake and decreased takeout meals [7], our intervention modeling focused on the effects of the app on BMI and assumed no other impacts on diet or physical activity.

Our model included the health system costs associated with changing disease prevalence and population longevity, which were calculated using an established protocol [40]. These costs were specific to the condition, age, and sex and were based on the timing of health events (first year of illness, subsequent years of illness, and the last 6 months of life). The change in the proportions of the population in each disease state resulted in proportional changes in health system costs and unrelated health system costs from people living longer. The intervention’s overall results reflect projected health gains and health system cost impacts over the remainder of the modeled population’s life course.

### Equity, Scenario, and Sensitivity Analyses

In addition to the main base case intervention, we conducted an equity analysis where an *equity adjustment* was applied in the model to eliminate differences in life expectancy between Māori (Indigenous) and non-Māori. Other scenario and sensitivity analyses were conducted to examine the potential impact of alternative modeling parameters and decisions. These consisted of the following: (1) the mass media campaign’s design is enhanced, leading to wider recognition of the campaign’s key message (68% recognition rather than 45%, using evidence from an evaluation of New Zealand’s Health Promotion Agency *Small Steps* campaign [58]); (2) improved weight loss apps that have been developed since the meta-analysis we used, leading to a hypothetical 50% improvement in app effectiveness (modeled by increasing the mean BMI reduction by 50%); (3) all app users use the app for more time to test impact if there is good long-term engagement (modeled using the pooled effect size for trial lengths of >3-9 months); (4a) weight regain is delayed by 1 year; (4b) weight regain is delayed by 5 years; (4c) no weight regain occurs throughout the remaining life of the modeled population (ie, a highly hypothetical scenario that quantifies the envelope of potential benefit that could be obtained if weight regain was avoided); (5) the effect size for weight loss app effectiveness that was used in the previous modeling by Cleghorn et al [36] is applied in this study’s updated model and intervention pathway; and (6) alternative discount rates of 0% and 6% are applied to health gains and healthy system costs to illustrate the impact of discount rates. This variation of discount rates is consistent with established health economic evaluation practices [41].

### Simulation Analysis

The model was built in Microsoft Excel and run using Ersatz (version 1.34; EpiGear International). Uncertainty around health gains and cost-effectiveness was quantified using a Monte Carlo analysis. The parameters were sampled independently 2000 times from each of their respective probability distributions. The presented results are the mean values, with 95% uncertainty intervals (UIs). The exception to this is the expected values in the scenario and sensitivity analyses, which did not include uncertainty analysis.

## Results

### Health Gain and Cost Savings

Across the remaining lifetime of the modeled 2011 New Zealand population, the mass media campaign to promote weight loss app use in the base case analysis had an estimated overall health gain of 181 (95% UI 113-270) QALYs and health care costs of –NZ $606,000 (–US $408,000; 95% UI –NZ $2,540,000 [–US $1,709,000] to NZ $907,000 [US $610,000]). The mean of the health care costs is negative, representing an overall savings to the health system and a cost-saving intervention. However, the 95% UI spans positive values, indicating that there remains a possibility that the intervention is not cost saving, albeit still cost-effective (ie, below the threshold of NZ $45,000 [US $30,000] per QALY gained, approximately the gross domestic product per capita for New Zealand that we use in our modeling [36]).

In the first 10 years after the intervention was implemented (2011-20), the mean health gain was 56 QALYs and net health system expenditures averaged NZ $850,000 (US $572,000) because of the cost of implementing that mass media campaign and few savings to the health system (although still below the cost-effective threshold of NZ $45,000 (US $30,000) per QALY gained). After 20 years (2011-30), the total health gain was 112 QALYs and the mass media campaign became cost-saving (–NZ $176,000 [–US $118,000]) for the health care system. Most of the health gain (62%) occurred between the years 2011 and 2030 (first 20 years after implementation), whereas most of the health system savings (71%) occurred after 2030 (ie, 20 years after implementation). The delayed health system savings was due to the initial up-front cost of implementing the intervention, which was also relatively high compared with the eventual reductions in downstream cost offsets because of altered disease rates.

Table 2 presents the results overall and by subpopulations over the remaining lifetime of the modeled population. The total health gains for non-Māori and Māori were 148 QALYs and 33 QALYs, respectively. Per 1000 population, this equated to 0.040 QALYS for non-Māori and 0.049 QALYS for Māori. When ethnicity was examined by sex and age group, the mean values consistently suggested greater per capita health gains for Māori. These mean values also suggest that the greatest health gain occurs among the age group 45-64 years and that, on average, the health gain was greater for men than for women. The exception to this was Māori women aged ≥65 years; this subgroup had a similar health gain to Māori men (0.079 QALYS per 1000 and 0.078 QALYS per 1000, respectively). Similar patterns were reflected in the health system cost estimates, which are, on average, savings to the health system. When the equity adjustment for Māori was applied (Table 3), the health gains for Māori increased to 40 QALYs and 0.060 QALYs per 1000. On the basis of the outcomes examined in this study, the modeled mass media campaign to promote weight loss apps among the general population would be expected to reduce health inequities arising from high BMI for Māori. However, the absolute reduction in health inequities would be small. In addition, this analysis assumed that the intervention would be as effective for Māori as it is for non-Māori (ie, the mass media campaign was culturally appropriate in design).

**Table 2:** Health gains and cost-effectiveness of a mass media campaign to promote smartphone apps for weight loss in New Zealand by age, sex, and ethnicity (lifetime impacts and 3% discount rate).

Sex, ethnicity, and age group (years)				Health gain in QALYs^a^ (95% UI^b^)		Health gain in QALYs per 1000 population (95% UI)		Health system costs^c^, NZ $ (US $; 95% UI)
All				181 (113 to 270)		0.041 (0.026 to 0.061)		–606,000 (2,540,000 to –907,000); 408,000 (–1,709,000 to 610,000)
Non-Māori, all ages				148 (85 to 231)		0.040 (0.023 to 0.062)		–491,000 (2,310,000 to 921,000); –330,000 (–1,555,000 to 620,000)
Māori, all ages				33 (18 to 53)		0.049 (0.027 to 0.079)		–115,000 (–494,000 to 158,000); –77,400 (–332,000 to 106,000)
**Men, all ages**				97 (48 to 170)		0.045 (0.022 to 0.079)		–436,000 (–1,872,000 to 554,000); –293,000 (–1,260,000 to 373,000)
	**Non-Māori^d^**							
		25-44	19		0.038		–85,000 (–57,200)	
		45-64	46		0.094		–556,000 (–374,000)	
		≥65	16		0.063		–127,000 (–85,500)	
	**Māori^d^**							
		25-44	6		0.080		–51,000 (–34,300)	
		45-64	9		0.171		–118,000 (–79,400)	
		≥65	1		0.078		–13,000 (–8750)	
**Women, all ages**				84 (44 to 141)		0.037 (0.020 to 0.063)		–170,000 (–1,370,000 to 698,000); –114,000 (–922,000 to 470,000)
	**Non-Māori^d^**							
		25-44	16		0.031		–45,000 (–30,000)	
		45-64	35		0.069		–369,000 (–248,000)	
		≥65	17		0.055		–77,000 (–52,000)	
	**Māori^d^**							
		25-44	6		0.066		–49,000 (–33,000)	
		45-64	9		0.143		–106,000 (–71,000)	
		≥65	1		0.079		–11,000 (–7000)	

^a^QALY: quality-adjusted life year.

^b^UI: uncertainty interval.

^c^A negative cost indicates that the intervention is cost-saving to the health system.

^d^The 95% uncertainty intervals for QALY and health system costs were not calculated for these subgroups.

**Table 3:** Results for Māori with equity adjustment applied (lifetime gains and 3% discount rate).

Population	Health gain in QALYs^a^ (95% UI^b^)	Health gain in QALYs per 1000 population (95% UI)	Health system costs^c^, NZ $ (US $; 95% UI)
All	40 (23 to 65)	0.060 (0.034 to 0.097)	–132,000 (–513,000 to 152,000); –89,000 (–345,000 to 102,000)
Men	20 (9 to 39)	0.062 (0.026 to 0.118)	–67,000 (–341,000 to 115,000); –45,000 (–229,000 to 77,000)
Women	20 (9 to 37)	0.058 (0.025 to 0.109)	–65,000 (–331,000 to 111,000); –44,000 (–223,000 to 75,000)

^a^QALY: quality-adjusted life year.

^b^UI: uncertainty interval.

^c^A negative cost indicates that the intervention is cost saving to the health system.

The modeled lifetime health gains among adults experiencing overweight or obesity were 0.065 (95% UI 0.041-0.097) QALYs per 1000 target population. By ethnicity, the gains for non-Māori were 0.064 (95% UI 0.037-0.100) QALYs and for Māori 0.070 (95% UI 0.038-0.113) QALYs.

### Scenario and Sensitivity Analyses

A range of results for scenario and sensitivity analyses are presented in Table 4. The modifications to the intervention pathway scenarios of either a more effective mass media campaign (eg, with better reach and targeting; scenario 1), more effective apps (scenario 2), or greater adherence to app use (scenario 3) all resulted in higher health gains and greater savings to the health system. When the model was altered to delay weight regain by 1 year (scenario 4a), 5 years (scenario 4b), or a highly hypothetical scenario of eliminating weight regain altogether (scenario 4c), the improved health gains and cost savings went from modest (eg, absolute increase in 20 QALYs for weight regain by 1 year) to markedly greater (eg, absolute increase in 14,544 QALYs for no weight regain). This highlights the substantial further health and economic benefits if weight regain was prevented across the remaining life course. The meta-analysis value [17] used in the previous modeling of mass media campaigns to promote weight loss [36] (scenario 5) yielded lower health gains and cost the health system, suggesting that apps have become increasingly more effective in weight management. The varied discount rates yielded results in the expected directions (scenarios 6a and 6b).

**Table 4:** Sensitivity and scenario analyses for a mass media campaign to promote weight loss smartphone apps by age, sex, and ethnicity (expected value analysis, lifetime perspective, and 3% discount rate, unless otherwise stated).

Sensitivity and scenario analyses^a^	Health gain in QALYs^b^	Difference in QALYs from base case, %	Health system costs^c^, NZ $ (US $)	Difference in health system costs from base case, %
Base case analysis	183	—^d^	–625,000 (–421,000)	—
1. Mass media campaign: higher recognition at 68%	276	51	–2,414,000 (–1,620,000)	286
2. Increase effect size of app use by 50%	274	50	–2,375,000 (–1,600,000)	280
3. 100% of population use the app for more time	278	52	–2,454,000 (–1,650,000)	293
4a. Delaying weight regain by 1 year	203	11	–2,400,000 (–1,620,000)	284
4b. Delaying weight regain by 5 years	1,261	589	–21,271,000 (–14,300,000)	3305
4c. No weight regain	14,727	7948	–286,465,000 (–193,000,000)	45,762
5. Value from the previous Cleghorn et al [36] mobile health modeling study	69	–62	1,549,000 (–1,040,000)	–348
6a. 0% discount rate	334	83	–1,892,000 (–1,270,000)	203
6b. 6% discount rate	114	–38	186,000 (–125,000)	–130

^a^Expected values given for all scenarios.

^b^QALY: quality-adjusted life year.

^c^A negative cost indicates that the intervention is cost saving to the health system.

^d^Base case is the reference with which scenarios are compared.

## Discussion

### Principal Findings and Interpretation

The results from this updated health economic simulation modeling suggest that a hypothetical government-initiated mass media campaign to promote use of smartphone weight loss apps would result in modest health gains over the remaining lifetime of the New Zealand adult population. There was an estimated net saving to the health care system because of reductions in BMI-related diseases, although the UIs included estimates that were cost-effective (rather than cost saving). The intervention would be expected to generate greater per capita health gain for Māori and therefore potentially reduce health inequities attributable to BMI differences between Māori and non-Māori, assuming that the intervention would be as effective for Māori as it is for non-Māori.

Several key characteristics contributed to this modeled mass media intervention being cost-effective. First, and perhaps most importantly for this study, recent evidence from a meta-analysis of randomized controlled trials and case-control studies shows that the use of smartphone weight loss apps largely results in some degree of weight loss, even when accounting for variations in the duration of app use [46]. Several different behavioral theories suggest that self-monitoring progress toward a goal is a critical step between setting a goal and achieving a goal [59]. Apps can be a useful tool for self-monitoring dietary behaviors [9]. Monitoring progress helps to ensure that goals are translated into action, and the effect of an intervention on goal attainment is mediated by the frequency of monitoring [59]. Second, there was a sizable target population for this intervention: adults with excess weight who own a smartphone make up approximately 60% of the population. Accordingly, even small reductions in weight can achieve wide-reaching benefits in health [1]. Third, government-initiated mass media campaigns are relatively low cost, especially in comparison with measures such as health service provision [60]. When designed well, mass media campaigns are effective at reaching a substantial proportion of the population with key health messages that can lead to modifications in behavior [29]. The World Health Organization has identified mass media as playing an important role in coherent national strategies for obesity prevention and management [61].

We found that there was very limited research examining whether mass media campaigns stimulate the specific action of adopting use of a smartphone app. Therefore, we relied on a UK evaluation of a mass media campaign that encouraged the use of a physical activity app [45]. A mass media campaign to promote a weight loss app would have been more appropriate. The tobacco control literature reports that media campaigns are associated with increased downloads of health apps (eg, smoking cessation apps) [62] and behavior changes further downstream (ie, attempts to quit smoking) [63]. There may be ways of making the mass media campaign more effective by targeting specific population groups in the messaging. For instance, there is evidence that mass media campaigns can better reach ethnic minorities using highly feasible social media or mass media that have reach to particular audiences [64] (eg, iwi radio and Māori Television in the New Zealand context).

### Comparison With Prior Work

This paper provides new evidence using updated parameters on the potential health gain and cost-effectiveness of this health intervention. The previous Cleghorn et al [36] modeling conducted by some of our team found that there was very minimal health gain from the mass media campaign to promote mHealth interventions and that the intervention was not cost-effective [36]. However, there are 3 notable differences between the methods used in this study and the previous modeling by Cleghorn et al [36]. Most importantly, in this study, we used the results of a very recent meta-analysis of studies of smartphone weight loss apps showing a meaningful decrease in BMI (–0.454 kg/m^2^, 95% CI –0.787 to –0.121 kg/m^2^) [46]. The previous modeling used the best available evidence at the time, which was a meta-analysis of mobile device interventions that included apps as well as tools such as SMS text messaging [36]. The pooled effect size, which is measured in kilograms rather than BMI units, showed a comparatively smaller reduction in weight (–0.430, 95% CI –0.609 to –0.252 kg); this smaller effect size was the main contributor to the difference between the 2 studies. For some intervention pathway steps that were the same between the 2 modeling studies, we used different, more recent data. Specifically, smartphone ownership was higher than previously modeled and the reach of the mass media campaign was lower. We also conceptualized some of the pathway between the initial intervention (ie, the mass media campaign) to the effect size (ie, the effect of weight loss app use on BMI) based on new evidence that was incorporated into other modeling on mass media campaigns [37]. In our sensitivity analyses, we used the previously modeled effect size to isolate how much of a difference was due to the revised pathway versus the effect size. We found that most of the improved outcomes in our modeling were due to greater effectiveness of the apps, with only a small proportion of the gain due to the different conceptualization of the pathway. Our study adds to the very limited simulation modeling evidence on the cost-effectiveness of health apps [36-38].

### Strengths and Limitations

A strength of this research is that it builds upon an established model [40], uses an effect size from a recent meta-analysis [46], and uses high-quality disease data that includes ethnicity-specific data [40]. There are a number of other ways that the mass media campaign could have been modeled, including alternative pathways that reflect numerous theories used to inform the development of mass media campaigns [29]. To account for a degree of uncertainty in input parameters and stochastic uncertainty, we modeled distributions of these inputs and quantified this variation using UIs. Parallel pathways for achieving impacts could be part of this intervention. For instance, we did not model that app uptake may also be by people who did not see the campaign but whose family members or friends saw it, started to use the app, and encouraged them to use it. In addition, the modeling might have underestimated the health benefits, given that there is evidence that mHealth interventions can result in changes in dietary intake beyond reduced weight loss, such as increased fruit and vegetable intake and decreased takeout meals [7] (which are typically high in sodium, sugar, and saturated fats). Some of these apps can also promote physical activity, which provides benefits to health beyond just a BMI pathway (eg, to mental health and cardiovascular health). It is unclear what proportion of the New Zealand population is already using a weight loss app and thus would not take up this intervention. However, despite the relatively high availability of mHealth apps, the level of awareness of such apps by individuals may be relatively low [65] and the apps being used may not be on the high-quality end of the spectrum. Our modeling did not account for differential impacts of either different apps or individual characteristics that could influence the adoption and engagement of apps, including the duration of this engagement and the quality of this engagement. Such differential impacts are too complex for inclusion in models of this design and also require highly detailed data for accurate measures. There is also no consistent definition for measuring constructs such as adherence to app use, which can encompass characteristics such as frequency, consistency, and detail of dietary monitoring [66].

### Potential Policy Implications

As part of a wide range of interventions to address the obesogenic environment and unhealthy dietary patterns, governments should consider investing in promoting such weight loss apps, along with funding research that improves their effectiveness and uptake in the community. But all such interventions should also be well evaluated, particularly given the large potential for scalability. Laws and taxes can create a less obesogenic environment (and have been shown to be more cost-effective than nutrition mass media campaigns [60]). However, such actions can take time, and there is political and food industry resistance [67]. Hence, there is a role for tools (such as smartphone apps) that support using dietary changes that improve weight management as complementary measures. Smartphone apps can also be combined with traditional interventions (eg, face-to-face counseling) [68], can form a component of a broader national social marketing health strategy [69], or they can be used as stand-alone treatments [54]. There may be further benefit from smartphone apps when face-to-face contact with patients must be limited [9], as in the case of the COVID-19 pandemic.

### Conclusions

Using recent evidence on the effectiveness of smartphone weight loss apps, a modeled mass media campaign to encourage the adoption of smartphone apps to promote weight loss among the New Zealand adult population is expected to yield an overall gain in health and to be cost saving to the health system. This is an update of previous modeling that showed a smaller health gain and that the intervention was not cost-effective. Although other interventions in the nutrition and physical activity space are even more beneficial to health and cost savings (eg, pricing policies and food reformulation [60]), governments may choose to include strategies to promote health app use as a feasible complementary measure.

## Appendix

Multimedia Appendix 1 Calculating the weighted pooled intervention effect size.

## Abbreviations

mHealth: mobile health
NSW: New South Wales
QALY: quality-adjusted life year
UI: uncertainty interval
