# Supplementary material for: The Cost-effectiveness of a Mass Media Campaign to Promote Smartphone Apps for Weight Loss: Updated Modeling Study
Source: JMIR Form Res. 2022 Apr 19;6(4):e29291. doi: 10.2196/29291 (PMC9066337; doi:10.2196/29291)

**Multimedia Appendix 1. Calculating the weighted pooled intervention effect size.**

This Multimedia appendix provides a description of how the weighted pooled intervention effect size was calculated. We combined evidence on the erosion of weight loss app use over time by Carter et al [54] and a meta-analysis by Islam et al on the effectiveness of weight loss apps at reducing BMI [46].

The study by Carter et al was a pilot randomized controlled trial in the United Kingdom to test the acceptability and feasibility (including recruitment, dropout, and adherence) of a trial that compared a smartphone app intervention (My Meal Mate) with two other weight management interventions (website or paper diary). In this trial, 43 people were randomized to the smartphone intervention, of which three dropped out. Participants were told to use the app every day for a week, and then to use it as much as they desired over the trial period (six months). Accordingly, the participants were not required to use the app in this study.

The study’s Figure 4 graphs the number of participants with the total number of days with complete dietary self-monitoring (not necessarily consecutive) for each of the three interventions. A complete day of dietary self-monitoring consisted of recording >500 kcal, but ≤5,000 kcal. Since this data was not reported numerically in the study, we used a free online software called Web Plot Digitizer to deter the numerical value of the plotted data points on this graph. We used these values to determine what proportion of the respondents had used the app for a duration of 3 months or less and what proportion had used the app for greater than three months (maximal usage of 6 months as per the trial’s length). For this graph, we extracted that 20 participants (46.5% of the sample used the app for 3 months (93 days). Compared to other published literature, this adherence rate is similar (ie, Turner-McGrievy et al found that at week 10 [70 days] 50% of participants were still meeting criteria for adherence to a mobile dietary app [19]).

In our modeling we assumed that, of the New Zealand population that downloaded and used the app, 53% of them would use the app for 3 months or less. Nearly half (47%), of users were assumed to adhere to app usage for greater than 3 months.

The next step in preparing the intervention effect size for modeling was to consult a systematic review and meta-analysis by Islam et al [46]. The authors included randomized controlled trials and case-control studies that compared smartphone weight loss app interventions with a control group. In their systematic review and meta-analysis, Islam et al sub-grouped the included studies based on the duration of the mobile app intervention (≤3 months; >3 months) and performed meta-analysis on each of these two subgroups. The authors did this for the outcome of weight, but not for BMI, which was one of their secondary outcomes.

We used the same division based on intervention duration (≤3 months; >3 months) to sub-group the studies reporting BMI outcomes that were included in the Islam et al review. We then performed a meta-analysis of each of these two subgroups using RevMan. We categorized the included BMI outcome studies by study duration using the information reported by Islam et al in their Table 1 (variation ‘study duration’). For the ≤3 months, one study had a duration of 6 weeks; one study had a duration of 2 months and the other two studies had durations of 3 months. For the >3 months, three studies lasted 6 months, one study was 7 months, and 2 studies lasted 9 months.

The result of our meta-analysis was -0.219 (95% CI -0.672, 0.223) for ≤3 months and -0.609 (-1.072, -0.146) for > 3 months (Figure 1, Figure 2). As demonstrated in the forest plots, there was substantial variation in the effectiveness of each trial which did not necessarily correspond to the duration of the trial. For instance, the 7 month study mean effect size was smaller than the three 6 month effect sizes. One of the 9 month mean effect sizes was same or less than the effectiveness of the 6 month effect sizes. We performed a sensitivity analysis where we excluded the three studies with a duration longer than 6 months to see if this reduced the effect size. The result was actually a greater weight loss, significant, but with wider confidence intervals: -0.829 (-1.500, -0.158).

As detailed in the main manuscript, the evidence from Carter et al and Islam et al were combined to calculate a pooled effect size that accounted for variations in adherence rates and degree of BMI loss.

Multimedia Appendix Figure 1. Meta-analysis of Islam et al studies for BMI outcome by intervention duration


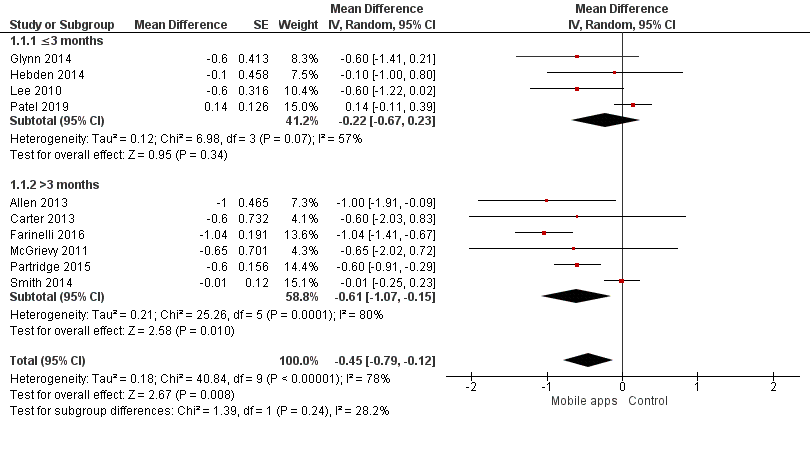


Multimedia Appendix Figure 2. Meta-analysis of Islam et al studies for BMI outcome by intervention duration, excluding >6 months study duration


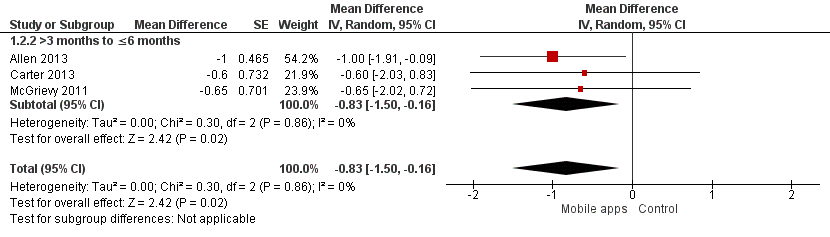

Supplement: Multimedia Appendix 1 [file formative_v6i4e29291_app1.docx]
